# Supplementary material for: Information provision and decision-making in the treatment of abdominal aortic aneurysm: A qualitative study of patient experience
Source: PLoS One. 2023 Oct 23;18(10):e0293354. doi: 10.1371/journal.pone.0293354 (PMC10593210; doi:10.1371/journal.pone.0293354)
Supplement: Attachment — Submitted filename: plos one.doc [file pone.0293354.s001.doc]

It is not specified after how long the patients were interviewed. It would be interesting to know because the elapsed time influences the memory.The work speaks not so much of the quality of the information provided, but of how much patients remember.

however, it is interesting to know what patients think about the information received. It is also interesting that they reported the dialogues they had with patients, but the sample is too limited.

I believe that these considerations expressed should be brought within the limits of the study
