# Supplementary material for: Information provision and decision-making in the treatment of abdominal aortic aneurysm: A qualitative study of patient experience
Source: PLoS One. 2023 Oct 23;18(10):e0293354. doi: 10.1371/journal.pone.0293354 (PMC10593210; doi:10.1371/journal.pone.0293354)
Supplement: Attachment — Submitted filename: Response to reviewers - Lecouturier.docx [file pone.0293354.s002.docx]

Reviewer #1: This is an interesting qualitative study examining shared medical decision making (SDM) in the context of abdominal aortic aneurysm (AAA). The authors recruited 15 patients (8 post surgery, 7 making a decision regarding intervention) and conducted interviews as well as reviewed recordings of medical consultations when available for those making a decision. Several themes were identified, and suggestions were made in terms of improvement in the SDM process. Particularly compelling were the data that compared the recordings of the medical consultations with the information provided by patients during their interviews with the investigators. My specific comments follow:

Major Comments:

1. The area of SDM is important and research in this area is growing and improving. Interventions regarding AAA provide a compelling and fertile area for this research. As such, this manuscript addresses an important area of medical practice and does so in an appropriate and particularly interesting context.

2. The manuscript is generally well-written with minor exceptions (listed below). The patient accounts are absorbing and enlightening.

3. Though there is much information presented, and I found all of it engaging, I thought the primary findings/recommendations could stand out a bit more. I’m not sure the correct format (or wording) for this but I found myself at the end of the manuscript retaining a great deal of information but not quite sure how the authors would prioritize it. I don’t intend to imply that this information is absent from the manuscript, and it is quite possible that I just missed it in my reading, but I had a hard time determining the most important conclusions that the work presented both in terms of clinical application and subsequent research focus.

*We thank the reviewer for their very helpful comments. We have amended the penultimate and final paragraph of the Discussion section and we hope this now more clearly states the conclusions from this research for the reader and its contribution to the field of SDM.*

4. The manuscript clearly makes a contribution to the field of SDM but I am not certain of the strength of that contribution. Perhaps this is related to #3 above.

*Please see response to point 3.*

5. I think more development of the issues surrounding what an objective review of the consultation recording revealed vs. what the patients heard/remembered from it would increase the strength of the contribution of this manuscript. Clearly this is a key area of concern and one that is likely to be ground zero for miscommunications, or at least misremembering of communications, between the parties involved.

*We have added more text to the Discussion to draw out and summarise the differences between the consultation and interview data.*

Minor Comments:

1. Throughout the document terms such as "a small number" and "several" are used. Please present the exact number.

*We understand that the use of terms such as ‘several’ can be frustrating for some readers. However, it is not customary in qualitative research to provide the exact numbers which is more in line with a quantitative paper. One disadvantage of using numbers is that they can, as Maxwell states, infer ‘greater generality for the conclusions than is justified, by slighting the specific context within which this conclusion is drawn. A particular setting or sample may be unrepresentative, and a facile reading of quantitative results may lead a reader to ignore this limitation.’* (Maxwell 2010)

*Maxwell JA. Using numbers in qualitative research. Qualitative inquiry. 2010 Jul;16(6):475-82.

2. There are several minor typos that should be corrected.

*All authors have read through the manuscript and hope we have found and corrected the minor typos.*

**3. When endovascular repair is first mentioned please provide the (EVAR) abbreviation so that this is then clear in Table 2. Probably readers who study SDM in areas apart from AAA will be interested in the manuscript and may not be aware of the initialism.**

*Thank you for drawing our attention to this. We have made this change.*

**4. In the research design sentence, suggest adding "a" between "was" and "qualitative" and suggest deleting "more" given that there is no referent for the implied comparison.**

*We have made these changes.*
 **5. I didn't understand what was meant by the sentence in the last line of the Identification of patients section - it begins "Given this opportunistic...."**

*Thank you for this comment. Patients in Group 2 (not yet had surgery) were identified by the clinical team as eligible after discussion by the Multi-Disciplinary Team. The clinical team did not record the numbers they approached who declined to participate. We have taken out this sentence as it refers only to Group 2 and, as the reviewer states, is confusing to the reader.*

**6. I was unclear what the "OPTION categories" were in the analysis section.**

*OPTION – Observing patient involvement (Research Version page 89) is a 12 item instrument to measure patient involvement in consultations. We have added this to the manuscript.*

**7. In the tables it was unclear to me what "Incidental" meant.**

*We have added a footnote to Table 3 to explain the term ‘incidental’.*

**Reviewer #2: it is not specified after how long the patients were interviewed. It would be interesting to know because the elapsed time influences the memory.**

*Group 1 were interviewed within three months of their procedure and Group 2 within five days of their last consultation. We have added this to the manuscript.*

**The work speaks not so much of the quality of the information provided, but of how much patients remember.** H**owever, it is interesting to know what patients think about the information received. It is also interesting that they reported the dialogues they had with patients, but the sample is too limited. I believe that these considerations expressed should be brought within the limits of the study**

We thank the reviewer for their comments. We have drawn attention to the limited sample size in the Strengths and Limitations section.
